# Curricular advances to develop medical students’ knowledge, skills, and attitudes in health advocacy

**DOI:** 10.1186/s12909-026-09837-w

**Published:** 2026-07-03

**Authors:** Nupur Agrawal, Rashmi Mullur, Hilary Koenig Whalen, Cambria Garell, Candace M. Gragnani, Emily Hotez, Annabelle J. Lee, Nicholas J. Jackson, Peter A. Quiros, Whitney Arnold

**Affiliations:** 1 Departments of Medicine and Pediatrics, David Geffen School of Medicine at the University of California Los Angeles, Los Angeles, CA USA; 2 Department of Medicine, David Geffen School of Medicine at the University of California Los Angeles, Los Angeles, CA USA; 3 David Geffen School of Medicine at the University of California Los Angeles, Los Angeles, CA USA; 4 Department of Pediatrics, David Geffen School of Medicine at the University of California Los Angeles, Los Angeles, CA USA; 5 University of California Los Angeles, Los Angeles, CA USA; 6 Department of Ophthalmology, David Geffen School of Medicine at the University of California Los Angeles, Los Angeles, CA USA; 7 Departments of Comparative Literature and Medicine, David Geffen School of Medicine at the University of California Los Angeles, Los Angeles, CA USA

**Keywords:** Health advocacy, Advocacy education, Undergraduate medical education, Competency-based education, Assessment, Evaluation

## Abstract

**Background:**

The American Medical Association’s Declaration of Professional Responsibility is an important covenant obligating present-day physicians to transcend personal beliefs and affiliations to advocate for changes that alleviate suffering and promote human health. Despite this recognition of advocacy as a pillar of physician duty, advocacy training in undergraduate medical education remains variable across institutions, elective in nature, and often insufficient in developing trainees’ professional identities and competence as health advocates.

**Methods:**

Educators at the UCLA David Geffen School of Medicine designed and delivered a novel half-day session required for all first-year medical students to nurture their professional identities as health advocates and empower them with knowledge, skills, and attitudes (KSA) needed to give voice to issues impacting the patients and communities they serve. Students attended a shared didactic followed by two focused workshops. The session was conducted annually from 2023–2025. Pre- and post-session data was collected after each iteration to evaluate changes in KSA.

**Results:**

A total of 509 first-year medical students participated in the session from 2023–2025 with 477 (94%) partaking in the session synchronously. All 509 participants (100%) completed at least one survey, and 361 (71%) completed both pre- and post-surveys. Pooled data showed that participation led to statistically significant improvement on a 7-point Likert scale at similar rates in all three cohorts in each of the measured domains: knowledge (Δ1.45, p < 0.001), skills (Δ1.37, *p* < 0.001), and attitudes (Δ0.44, *p* < 0.001).

**Conclusions:**

A required half-day advocacy session supported trainees in developing their professional identities and acquiring foundational KSA needed to fulfill their roles as health advocates. Implementing this session across institutions can broaden its impact. Longitudinal data collection can clarify its long-term influence on physician engagement in advocacy and on health outcomes of patients and communities.

**Supplementary Information:**

The online version contains supplementary material available at 10.1186/s12909-026-09837-w.

## Background

Every year, as over 20,000 students matriculate into medical schools across the United States, educators are tasked with preparing the next generation of physicians to meet society’s changing health needs in the context of increasingly complex healthcare delivery systems [1–5]. Multiple accrediting and medical organizations emphasize teaching medical students the foundational skills needed to improve health outcomes at both the individual and population levels, ideally prioritizing whole health, patient and physician well-being, and using integrated approaches grounded in health systems science, population health, and public health [6–9]. Accordingly, in addition to developing strong clinical acumen, students must develop a broader set of professional skills they can leverage to meaningfully impact the health of the patients and communities they serve [1, 2, 10, 11]. Educators can help students achieve this goal through training in health advocacy [12].

The American Medical Association (AMA) Declaration of Professional Responsibility emphasizes that concerted and committed effort is needed to improve human health globally and positions advocacy as a core aspect of present-day physicians’ duties, urging physicians to transcend personal beliefs and affiliations to advocate for changes that promote human health [13]. Cultivating this aspect of professional identity requires thoughtful curricula that teach practical advocacy-related knowledge, skills, and attitudes (KSA) that students can use to identify and critically evaluate areas for improving healthcare delivery and outcomes.

Despite the clear recognition of its importance, advocacy training in undergraduate medical education (UME) remains variable and challenging [1, 2, 4, 14–17]. Currently, over three-fourths of American medical schools offer at least one course in advocacy, but courses are often elective in nature, created in silos, have varying targeted competencies, and are infrequently published as shared curricula [14, 18, 19]. Understanding key elements of existing advocacy courses can help identify best practices, persistent gaps, and opportunities to strengthen advocacy training in UME.

For example, in 2013, the University of Chicago Pritzker School of Medicine integrated advocacy content into its required Health Care Disparities course for first-year medical students to circumvent the issue of students only electively receiving exposure to advocacy and social responsibility concepts [20]. Activities included lectures from invited experts, seminars, essay assignments, and small-group community-based projects. Pre- and post-surveys showed that participation increased the percentage of students who considered themselves to be advocates, but the authors noted the need for greater skills-based training. Faculty at Rutgers New Jersey Medical School later described their longitudinal Health Equity and Social Justice thread that explicitly emphasized physician engagement and advocacy [21]. Strengths included multifaceted student involvement and intentional faculty and resident development efforts. Outcomes showed improved learner preparedness to recognize bias and work with disadvantaged communities, while the authors recognized limitations related to assessment of skills and long-term outcomes. At Wake Forest University School of Medicine, students and faculty co-founded an elective Health Justice Advocacy Certificate Program that includes didactic lectures, journal club, elective activities, and a capstone project and explicitly encourages practical application of lessons learned though formal evaluation remains pending [3].

Collectively, these curricula demonstrate multiple innovative approaches to advocacy education while also underscoring lack of curricular standardization across institutions [1, 3, 16, 19, 22]. Published evaluations often focus on learner satisfaction and attitudes rather than measurable changes in advocacy-related knowledge and skills related to curricular engagement [11, 15, 22–24].

Herein we describe a novel UME advocacy session and report the results of our multi-year, quasi-experimental, single-institution analysis of pre- and post-session survey data. Our findings elucidate successful strategies for improving learners’ knowledge retention, skill development applicable to clinical work, and attitudes toward health advocacy that align with professional responsibilities. To our knowledge, this is one of the largest studies done to date providing robust pre- and post-intervention data on medical students’ advocacy-related KSA [25–28]. We hope our work will stimulate broader conversations at medical schools nationally and internationally regarding the importance of integrating advocacy training early into medical education, provide educators with practical tools to implement successful curricula in their programs, and contribute to improved societal health outcomes long-term.

## Methods

Medical educators at the University of California Los Angeles (UCLA) David Geffen School of Medicine (DGSOM) created a novel 3.5-hour evidence-informed standalone advocacy session in 2023 after students and faculty expressed that an hourlong didactic on health advocacy delivered the year prior was insufficient and that a more comprehensive training program was both desired and necessary. This need was identified through student course evaluations and faculty efforts to broaden advocacy training in alignment with priorities of DGSOM’s new HEALS (Healer, Educator, Advocate, Leader, Scholar) curriculum. HEALS aims to equip students with relevant, cutting edge-tools needed to address the challenges physicians face in providing care to patients in a rapidly evolving and increasingly complex healthcare environment, with clear emphasis on early training to support the development of students’ identities and skills as health advocates.

We thus embedded our required session into the Foundations of Practice (FOP) course for first-year medical students, which provides a longitudinal and creative space for trainees to develop KSA needed to provide exemplary clinical care to diverse patient populations and affect meaningful changes in healthcare. Students’ learning in FOP is generally assessed through Objective Structured Clinical Examinations, written exams, formative evaluations by small group instructors, and self-assessment. For our session, we provided two questions for the written exam as well as opportunities for additional self-assessment through the pre- and post-survey. We purposefully scheduled our session during the last quarter of the year-long FOP course to provide students with a culminating opportunity to reflect upon the healthcare delivery challenges and opportunities to which they were introduced in the first three quarters of the year.

### Session structure

Our advocacy session had several overarching goals: (1) to nurture the development of students’ professional identities as health advocates; (2) to empower them with the KSA needed to give voice to important issues they identify in caring for patients and communities; (3) to facilitate their ability to enact positive change across individual, community, and system levels; (4) to encourage career-long engagement in advocacy; and (5) to improve patient and community health outcomes over time. We did not promote any particular advocacy views or agendas and instead focused on cultivating lifelong critical thinking skills. Our ultimate goal was to create an educational model that could be adapted across programs and institutions if results of robust evaluation found the session to be effective [1, 3, 15, 19, 22].

**Fig. 1:**
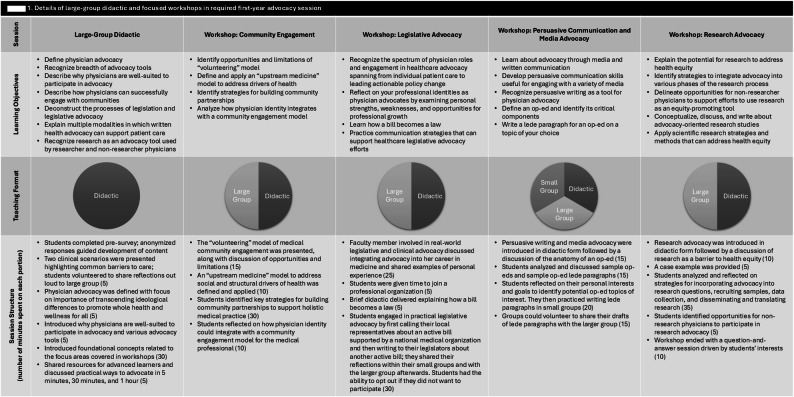
Details of large-group didactic and focused workshops in required first-year advocacy session

Evidence from literature detailing advocacy curricula in the undergraduate and graduate medical education spaces guided the integration of key features into the session, including the variety of teaching methods (large-group didactic and small group workshop) and the four workshop topics: community engagement, legislative advocacy, persuasive communication and media advocacy, and research advocacy [22, 24]. Figure 1 describes the format, objectives, and content of each curricular component in detail. Core concepts of advocacy were introduced in an initial 50-minute didactic and then explored more deeply in the subsequent 65-minute small-group workshops, each led by 1–2 faculty members. Students were asked to select two of the four workshops to attend, given the 3.5-hour session time constraint. While attendance at all four workshops would have been ideal, we prioritized student depth of learning during the workshops, with breadth covered through the didactic, in which students were introduced to content around all four workshop topics. Attendance was softly capped at 60 per workshop for relatively even distribution of students across workshops. After attending the didactic, students referenced workshop descriptions and room locations via the learning management system. Instructors remained largely the same across the years and met immediately after each iteration to collectively discuss observations and next steps. Handoffs were provided when instructors changed.

Using self-determination theory as a conceptual framework, we ensured that objectives were clearly stated, learners had autonomy in indicating the focused workshops they wanted to attend, sessions prioritized the development of practical skills and relational and experiential learning over theoretical discussions, and educators emphasized the connections of the training to patient and community health outcomes [22, 29, 30]. Given the increasingly polarized broader sociopolitical climate, we embedded the most recent iteration of the session further into the apolitical calling of the AMA Declaration of Professional Responsibility for all physicians to advocate for the health and well-being of patients.

All curricular components were recorded for students to view the didactic and complete workshop activities asynchronously. Students unable to attend the session in-person in 2023 and 2024 completed the session online asynchronously the following week; due to overarching logistical changes, no asynchronous learning option was available in 2025. Aside from completion of the pre- and post-survey, no formal mechanisms were in place to verify completion of or monitor degree of engagement with the asynchronous workshop activities.

### Survey methodology

Drawing upon prior literature demonstrating learning gains following short-term curricular interventions, we prioritized robust evaluation of our 3.5-hour session to determine whether it effectively imparted advocacy-related KSA to students [19, 31]. There are no validated training survey tools to our knowledge in this space; accordingly, we adapted published surveys on MedEdPORTAL [32, 33]. The pre- and post-surveys were intentionally designed to look beyond trainee satisfaction and assess short-term changes in learner KSA. Knowledge, skills, and attitudes were operationalized through survey questions mapped to session objectives and assessed using a 7-point Likert scale. Knowledge items measured self-reported understanding, skills items reflected self-efficacy, and attitudes were measured using belief-based items. Pre- and post-surveys were purposefully non-identical to capture distinct constructs at baseline and following the intervention. The 27-question pre-survey focused on baseline familiarity with advocacy concepts, learner goals, and demographics, while the 34-question post-survey emphasized outcomes, including changes in KSA, perceived session impact, and reflective application. Our primary analysis focused on KSA items that aligned across both surveys.

One week prior to the session, students were given the link to two surveys using Qualtrics, an online survey software program approved by UCLA Health for safe and secure data collection. The first survey included brief details of the workshops, and students used it to indicate their preferences for which two of the four workshops they wanted to attend. The second survey was the pre-survey (Appendix 1). Students were given protected time in FOP to complete these surveys but had until the start of the session a week later to finish. At the end of the session, students received the link to the post-survey (Appendix 2); protected time was built into the second workshop for survey completion.

Informed consent was obtained from all participants with survey completion constituting consent for inclusion in the study. Participants were informed of the study’s purpose, the voluntary and confidential nature of their participation, and their right to withdraw at any time. This study was reviewed by our institutional review board and certified as exempt.

### Statistical analysis approach

Descriptive statistics were computed for baseline characteristics (in-person synchronous versus online asynchronous delivery, cohort, race, gender) and outcome measures (KSA domains), including means with standard deviation and percentages where appropriate. Domain internal consistency was measured using Cronbach’s alpha. Bivariate differences between those with complete data (completed surveys at both time points) and incomplete data (completed only pre- or post-survey) were examined using Welch’s t-test and chi-square. Mixed-effects linear regression models were used to examine change in KSA from pre- to post-survey, with random intercept for participant and random slope for time. Fixed effects were specified for time (modeled as a categorical factor with two time points). To accommodate missing data points without excluding entire cases, data from all participants with at least one timepoint was used in the mixed-effects model.

To explore differences in change over time between cohorts (2023 vs 2024 vs 2025) and delivery methods (in-person synchronous vs online asynchronous), the same mixed-effects models described above were used and incorporated interaction terms between time and cohort and, in separate models, between time and delivery. In addition, similar models were used to analyze the difference in change over time between students who did and did not attend the specific workshops. The mixed effects models employed are robust to missing data under a Missing at Random assumption when estimated using maximum likelihood. To further assess the impact of missing data on these analyses, a complete case sensitivity analysis was also conducted. Standardized effect sizes analogous to Cohen's d are reported based on the mixed effects modeling of normalized (i.e., z-scored) values.

All statistical analyses were conducted using R version 4.3.2. Mixed effects modeling was conducted using the *nlme* package. A two-sided *p* value < 0.05 was considered statistically significant.

## Results

A total of 509 first-year medical students, representing 97% of first-year matriculants, participated in the session across three years (168 students in 2023 representing 97% of the matriculated class, 168 in 2024 representing 95%, 170 in 2025 representing 96%). There was no statistical difference in the demographic characteristics of the three classes, including gender (*p* = 0.26) and race (*p* = 0.25), based on self-reported information collected in the pre-survey (Table 1). Data from all three classes was thus pooled.

**Table 1:** Demographic characteristics of cohorts

**Characteristic**	**Overall** (*N* = 509)	**Cohort 2023** (*n* = 168)	**Cohort 2024** (*n* = 168)	**Cohort 2025** (*n* = 173)	**p**^**1**^
Gender, *N (%)*					0.26
Female	257 (50.5%)	93 (55.4%)	75 (44.6%)	89 (51.4%)	
Male	158 (31%)	51 (30.4%)	52 (31%)	55 (31.8%)	
Nonbinary	5 (1%)	1 (0.6%)	2 (1.2%)	2 (1.2%)	
Other	2 (0.4%)	1 (0.6%)	0 (0%)	1 (0.6%)	
Prefer not to respond	87 (17.1%)	22 (13.1%)	39 (23.2%)	26 (15%)	
Race, *N (%)*					0.25
^ a^AIAN	3 (0.6%)	1 (0.6%)	0 (0%)	2 (1.2%)	
^ b^AAPI	143 (28.1%)	46 (27.4%)	39 (23.2%)	58 (33.5%)	
Black	46 (9%)	21 (12.5%)	15 (8.9%)	10 (5.8%)	
Hispanic	71 (13.9%)	27 (16.1%)	22 (13.1%)	22 (12.7%)	
Multiple/Other	46 (9%)	18 (10.7%)	18 (10.7%)	10 (5.8%)	
White	101 (19.8%)	32 (19%)	31 (18.5%)	38 (22%)	
Prefer not to respond	99 (19.4%)	23 (13.7%)	43 (25.6%)	33 (19.1%)	

^1^ Fisher’s exact test or Pearson Chi-Square

^a^American Indian/Alaskan Native

^b^Asian American/Pacific Islander

All 509 participants (100%) completed at least one survey, and 71% (*n* = 361/509) completed both pre- and post-surveys. Most students (94%) participated in the session synchronously in person (*n* = 477/509), while 6% (*n* = 32/509) participated asynchronously online (available only in 2023 and 2024). Forty-five percent of students (*n* = 229/509) attended the research workshop, 46% (*n* = 235/509) attended the community workshop, 36% (*n* = 184/509) attended the legislative workshop, and 22% (*n* = 113/509) attended the communication workshop.

### General knowledge, skills, and attitudes

Knowledge, skills, and attitudes scales showed high internal consistency with Cronbach’s alphas of 0.87, 0.72, and 0.84, respectively. Participation in the session led to statistically significant improvement at similar rates in all three classes in each of the measured domains: knowledge (Δ1.45, *p* < 0.001), skills (Δ1.37, *p* < 0.001), and attitudes (Δ0.44, *p* < 0.001) (Table 2). While all parameters improved, students showed greater absolute increase in acquisition of knowledge and skills. In complete case sensitivity analyses, the direction, magnitude, and statistical significance of the findings was unchanged.

**Table 2:** Changes from pre- to post-survey in knowledge, skills, and attitudes

	**Pre-Survey** Mean (95% CI) *N* = 457	**Post-Survey** Mean (95% CI) N = 413	**Change** Δ (95% CI)	**δ**^**1**^	**P**^**2**^
^a^Knowledge Scale	4.34 (4.24, 4.43)	5.78 (5.69, 5.87)	1.45 (1.34, 1.56)	1.18	<.001
* Q2 Definition*	5.13 (5.01, 5.24)	6.21 (6.12, 6.29)	1.08 (0.96, 1.21)	0.88	<.001
* Q7 State Reps*	2.89 (2.72, 3.05)	5.45 (5.29, 5.61)	2.56 (2.36, 2.76)	1.19	<.001
* Q8 Federal Reps*	3.22 (3.04, 3.41)	5.44 (5.28, 5.6)	2.22 (2.02, 2.42)	1.04	<.001
* Q11 Profess. Org*	4.6 (4.42, 4.77)	5.8 (5.68, 5.92)	1.2 (1.01, 1.4)	0.69	<.001
* Q14 Approaches*	3.74 (3.57, 3.91)	5.68 (5.54, 5.81)	1.94 (1.74, 2.13)	1.02	<.001
* Q15 Strategies*	4.65 (4.51, 4.8)	5.81 (5.69, 5.92)	1.16 (0.99, 1.32)	0.77	<.001
* Q16 Research*	5.6 (5.49, 5.7)	6.03 (5.93, 6.13)	0.44 (0.31, 0.57)	0.38	<.001
* Q17 Methods*	4.87 (4.73, 5.01)	5.81 (5.69, 5.93)	0.94 (0.78, 1.1)	0.64	<.001
^b^Skills Scale	4.36 (4.25, 4.47)	5.73 (5.64, 5.83)	1.37 (1.25, 1.49)	1.05	<.001
* Q6 Advocate*	5.38 (5.25, 5.51)	6.27 (6.17, 6.36)	0.89 (0.74, 1.03)	0.69	<.001
* Q9 Contact*	4.06 (3.87, 4.24)	5.84 (5.7, 5.98)	1.78 (1.58, 1.99)	0.9	<.001
* Q13 Lede Paragraph*	3.42 (3.24, 3.59)	5.28 (5.12, 5.45)	1.87 (1.67, 2.07)	0.93	<.001
* Q18 Experience*	4.59 (4.43, 4.75)	5.55 (5.41, 5.69)	0.96 (0.79, 1.13)	0.56	<.001
^c^Attitude Scale	5.72 (5.65, 5.79)	6.16 (6.08, 6.23)	0.44 (0.36, 0.51)	0.56	<.001
* Q1 Essential*	6.52 (6.45, 6.6)	6.51 (6.43, 6.59)	−0.02 (−0.1, 0.06)	−0.02	0.69
* Q3 Well-Suited*	6.11 (6.02, 6.21)	6.39 (6.3, 6.48)	0.28 (0.17, 0.38)	0.28	<.001
* Q4 Identity*	5.96 (5.86, 6.06)	6.19 (6.09, 6.28)	0.22 (0.13, 0.32)	0.2	<.001
* Q5 Confidence*	5.5 (5.39, 5.62)	6.08 (5.98, 6.18)	0.57 (0.45, 0.7)	0.48	<.001
* Q10 Comfort*	3.94 (3.76, 4.11)	5.48 (5.33, 5.62)	1.54 (1.35, 1.73)	0.81	<.001
* Q12 Writing*	5.9 (5.8, 6)	6.2 (6.11, 6.29)	0.3 (0.18, 0.41)	0.29	<.001
* Q19 Collaboration*	6.27 (6.18, 6.37)	6.35 (6.27, 6.44)	0.08 (−0.02, 0.18)	0.09	0.12
* Q20 Future*	6.15 (6.05, 6.25)	6.27 (6.18, 6.35)	0.12 (0.02, 0.22)	0.12	0.02
* Q21 Incorporation*	5.12 (4.98, 5.25)	5.93 (5.83, 6.03)	0.81 (0.66, 0.97)	0.61	<.001

^1^Cohen’s d standardized effect size

^2^Mixed-effects linear regression with participant random intercept and time random slope. Main effects for time.

^a^Average of 8 Knowledge Questions: I can clearly define physician advocacy. I know the names of my state congresspeople. I know the names of my federal congresspeople. I know at least one professional organization with which I can collaborate on advocacy efforts. I can distinguish between the volunteerism model and the “upstream medicine” approach to community engagement. I can identify key strategies for building community partnerships. I understand conceptually how research can be used as an advocacy tool. I am familiar with specific research methods a researcher can use to promote equity/social justice in studies

^b^Average of 4 Skills Questions: I know at least one way to advocate in less than 5 minutes. I know how to contact my representatives. I know how to write a lede paragraph for an op-ed. I have experience using research findings to promote social justice

^c^Average of 9 Attitude Questions: Advocacy work is essential for change. My profession makes me well-suited to be an advocate. Being an advocate is an important part of who I currently am. I feel confident in my ability to advocate for causes I care about. I feel comfortable contacting my representatives. Persuasive writing is a powerful tool for physician advocacy. Collaboration is foundational to the success of advocacy efforts. Being an advocate will be an important part of my future career. I have a clear understanding of how I would like to incorporate advocacy into my professional activities this coming year

When examining the individual items comprising the knowledge (k = 8), skills (k = 4), and attitudes (k = 9) scales, all items of the knowledge and skills scales and 7 of the 9 items in the attitudes scales showed reliable improvement after students participated in our advocacy session. Students reported being better able to define physician advocacy, understand that being a physician makes them well-suited to be an advocate, see the advocate role as an important part of their identity and their future career, feel confident in their ability to advocate for causes they care about, know ways to advocate in less than 5 min, and determine how they’d like to incorporate advocacy into their professional activities during their upcoming clerkship year (*p* < 0.001 for all questions).

Session participation did not lead to statistically significant change in attitudes around whether advocacy work is essential for change (Q1) and whether collaboration is foundational to the success of advocacy efforts (Q19). These two questions had the highest pre-survey scores (both above 6.25 on the Likert scale).

### Focus areas

We analyzed whether participation in the overall session led to acquisition of KSA in the four key areas of focus and whether there was differential change in these KSA based on workshop attendance (Table 3).

**Table 3:** Differential change over time in knowledge, skills, and attitudes by workshop attendance

		**Attended**				**Did not Attend**				
		**Pre-Survey **Mean (95%CI)	**Post-Survey**Mean (95%CI)	**Change** Δ(95% CI)	δ^1^	**Pre-Survey **Mean (95%CI)	**Post-Survey **Mean (95%CI)	**Change** Δ(95% CI)	δ^1^	p^2^
*Community Engagement*										
		N = 210	N = 236			N = 247	N = 177			
Knowledge Scale		4.31 (4.17, 4.45)	5.73 (5.61, 5.85)	1.42 (1.27, 1.57)	1.21	4.35 (4.22, 4.48)	5.84 (5.71, 5.98)	1.49 (1.33, 1.65)	1.17	0.52
^ a^*Q14*		3.84 (3.59, 4.08)	5.78 (5.6, 5.96)	1.95 (1.67, 2.22)	1.06	3.66 (3.43, 3.89)	5.54 (5.34, 5.75)	1.88 (1.6, 2.16)	0.97	0.75
^ b^*Q15*		4.7 (4.49, 4.91)	5.92 (5.77, 6.07)	1.22 (0.99, 1.45)	0.85	4.61 (4.42, 4.81)	5.66 (5.49, 5.84)	1.05 (0.81, 1.29)	0.67	0.3
*S*kill Scale		4.28 (4.12, 4.44)	5.59 (5.46, 5.71)	1.31 (1.14, 1.47)	1.03	4.43 (4.28, 4.58)	5.92 (5.78, 6.06)	1.49 (1.32, 1.67)	1.1	0.13
Attitude Scale		5.76 (5.66, 5.85)	6.13 (6.03, 6.23)	0.38 (0.28, 0.47)	0.53	5.69 (5.6, 5.78)	6.2 (6.08, 6.31)	0.51 (0.4, 0.62)	0.59	0.08
*Legislative Advocacy*										
		N = 156	N = 185			N = 301	N = 228			
Knowledge Scale		4.45 (4.29, 4.62)	5.89 (5.76, 6.03)	1.44 (1.27, 1.61)	1.17	4.27 (4.15, 4.39)	5.7 (5.58, 5.82)	1.43 (1.28, 1.57)	1.21	0.91
^ c^*Q11*		4.72 (4.42, 5.02)	6.07 (5.88, 6.25)	1.35 (1.03, 1.67)	0.76	4.53 (4.31, 4.75)	5.59 (5.43, 5.75)	1.06 (0.81, 1.31)	0.63	0.16
Skill Scale		4.57 (4.38, 4.75)	5.88 (5.73, 6.02)	1.31 (1.12, 1.51)	0.99	4.25 (4.12, 4.39)	5.63 (5.51, 5.76)	1.38 (1.22, 1.54)	1.11	0.59
^ d^*Q9*		4.46 (4.15, 4.78)	6.29 (6.1, 6.49)	1.83 (1.5, 2.16)	0.92	3.84 (3.61, 4.07)	5.49 (5.31, 5.66)	1.65 (1.39, 1.91)	0.91	0.4
Attitude Scale		5.76 (5.65, 5.88)	6.27 (6.16, 6.38)	0.51 (0.39, 0.62)	0.65	5.7 (5.61, 5.78)	6.07 (5.97, 6.17)	0.37 (0.28, 0.47)	0.47	0.07
^ e^*Q10*		4.18 (3.88, 4.48)	5.97 (5.77, 6.18)	1.79 (1.49, 2.1)	0.96	3.8 (3.58, 4.02)	5.09 (4.91, 5.27)	1.29 (1.05, 1.53)	0.7	**0.01**
*Persuasive Communication*										
		N = 94	N = 112			N = 363	N = 301			
Knowledge Scale		4.19 (3.98, 4.4)	5.83 (5.65, 6.01)	1.64 (1.41, 1.86)	1.27	4.37 (4.27, 4.48)	5.76 (5.65, 5.87)	1.39 (1.26, 1.51)	1.15	0.06
Skill Scale		4.38 (4.14, 4.62)	6.02 (5.84, 6.21)	1.64 (1.4, 1.89)	1.2	4.35 (4.23, 4.48)	5.63 (5.52, 5.74)	1.27 (1.13, 1.41)	0.99	**0.01**
^ f^*Q13*		3.6 (3.23, 3.98)	6.21 (5.92, 6.51)	2.61 (2.21, 3.01)	1.34	3.36 (3.17, 3.55)	4.94 (4.75, 5.12)	1.57 (1.35, 1.8)	0.79	**< .001**
Attitude Scale		5.67 (5.52, 5.81)	6.24 (6.09, 6.38)	0.57 (0.42, 0.71)	0.7	5.73 (5.66, 5.81)	6.12 (6.04, 6.21)	0.39 (0.3, 0.47)	0.5	**0.04**
^ g^*Q12*		6.04 (5.82, 6.25)	6.33 (6.15, 6.5)	0.29 (0.06, 0.53)	0.29	5.87 (5.76, 5.98)	6.16 (6.05, 6.27)	0.29 (0.16, 0.42)	0.28	0.99
*Research Advocacy*										
		N = 208	N = 228			N = 249	N = 185			
Knowledge Scale		4.32 (4.18, 4.46)	5.79 (5.66, 5.91)	1.47 (1.31, 1.62)	1.2	4.35 (4.22, 4.48)	5.77 (5.64, 5.91)	1.43 (1.27, 1.59)	1.17	0.72
^ h^*Q16*		5.72 (5.56, 5.88)	6.22 (6.08, 6.35)	0.5 (0.32, 0.68)	0.46	5.5 (5.35, 5.64)	5.81 (5.66, 5.96)	0.31 (0.13, 0.5)	0.27	0.16
^ i^*Q17*		4.9 (4.69, 5.1)	6.05 (5.89, 6.21)	1.15 (0.93, 1.37)	0.81	4.85 (4.66, 5.04)	5.51 (5.34, 5.69)	0.66 (0.43, 0.89)	0.44	**0.003**
Skill Scale		4.33 (4.16, 4.49)	5.68 (5.55, 5.81)	1.35 (1.18, 1.52)	1.04	4.39 (4.24, 4.54)	5.8 (5.66, 5.94)	1.41 (1.23, 1.58)	1.07	0.65
Attitude Scale		5.73 (5.63, 5.83)	6.11 (6.01, 6.22)	0.38 (0.28, 0.48)	0.47	5.71 (5.62, 5.8)	6.21 (6.1, 6.32)	0.5 (0.39, 0.61)	0.66	0.11

Statistically significant results (*p* < 0.05) are bolded

^1^Cohen’s d standardized effect size

^2^P value for the difference-in-difference estimate from a mixed-effects linear regression with participant random intercept and time random slope. Main effects for time, workshop attendance, and their interaction.

^a^Q14: I can distinguish between volunteerism model and “upstream medicine” approach to community engagement

^b^Q15: I can identify key strategies for building community partnerships

^c^Q11: I know at least one professional organization with which I can collaborate on advocacy efforts

^d^Q9: I know how to contact my representatives

^e^Q10: I feel comfortable contacting my representatives

^f^Q13: I know how to write a lede paragraph for an op-ed

^g^Q12: Persuasive writing is a powerful tool for physician advocacy

^h^Q16: I understand conceptually how research can be used as an advocacy tool

^i^Q17: I am familiar with specific research methods a researcher can use to promote equity/social justice in studies

#### Community engagement

Participation in the overall session led to improvement in students’ ability to distinguish the volunteerism model and “upstream medicine” approaches of community engagement (Q14: Δ1.94, *p* < 0.001) and identify key strategies for building community partnerships (Q15: Δ1.16, *p* < 0.001), Table 2. However, attendees of the community engagement workshop did not show any reliably different outcomes than non-attendees (Table 3).

#### Legislative advocacy

The overall session led to improvement in students’ ability to know the names of their state (Q7: Δ2.56, *p* < 0.001) and federal (Q8: Δ2.22, *p* < 0.001) congresspeople, know how to contact these representatives (Q9: Δ1.78, *p* < 0.001) and feel comfortable in doing so (Q10: Δ1.54, *p* < 0.001), and identify at least one professional organization with which they could collaborate (Q11: Δ1.2, *p* < 0.001), Table 2. Attendees of the legislative workshop reported being more comfortable contacting their representatives than non-attendees (Q10: Δ1.79 attendees vs Δ1.29 non-attendees, *p* = 0.01), Table 3.

#### Persuasive communication and media advocacy

Participation in the overall session led to increased recognition of persuasive writing as a powerful tool for physician advocacy (Q12: Δ0.3, *p* < 0.001) and knowledge of how to write a lede paragraph for an op-ed (Q13: Δ1.87, *p* < 0.001), Table 2. Attendees of the persuasive communication workshop reported greater improvement in overall skills (Δ1.64 attendees vs Δ1.27 non-attendees, *p* = 0.01), including in writing a lede paragraph (Q13: Δ2.61 attendees vs Δ1.57 non-attendees, *p* < 0.001), Table 3. They also had greater increase in overall attitudes (Δ0.57 attendees vs Δ0.39 non-attendees, p = 0.04).

#### Research advocacy

The overall session helped students better understand conceptually how research can be used as an advocacy tool (Q16: Δ0.44, *p* < 0.001), become more familiar with research methods to promote equity and social justice in research studies (Q17: Δ0.94, *p* < 0.001), and gain experience using research findings to promote social justice (Q18: Δ0.96, *p* < 0.001), Table 2. Participants of the research advocacy workshop reported being more familiar with specific research methods that promote equity and social justice than non-participating peers (Q17: Δ1.15 attendees vs Δ0.66 non-attendees, *p* = 0.003), Table 3.

### In-person synchronous versus online asynchronous learning

Table 4 examines the differences in synchronous and asynchronous learning. There were no reliable differences in the change in KSA domains between synchronous and asynchronous learning at the *p* < 0.05 level, but synchronous learners showed greater absolute improvement across all three domains—knowledge (Δ1.47 vs Δ1.14, *p* = 0.15), skills (Δ1.4 vs Δ0.98, *p* = 0.11), and attitudes (Δ0.45 vs Δ0.18, *p* = 0.07)—at *p* < 0.15. Notably, only synchronous learners had statistically significant improvement in attitudes (*p* < 0.001 synchronous vs *p* = 0.22 asynchronous), which was possibly due to slightly higher baseline attitude scores in asynchronous learners (mean = 5.98 asynchronous vs 5.71 synchronous) although this was not significant at the 0.05 level (*p* = 0.07). Given the small sample size (*n* = 32) participating asynchronously, these results should be considered exploratory in nature.

**Table 4:** Differential change over time in knowledge, skills, and attitudes by asynchronous delivery

	**Pre-Survey** Mean (95% CI)	**Post-Survey** Mean (95% CI)	**Change** Δ(95% CI)	**δ**^**1**^	**p**^**2**^
Knowledge Scale					
Synchronous, *N* = *[434, 381]*	4.33 (4.24, 4.43)	5.8 (5.71, 5.9)	1.47 (1.36, 1.58)	1.21	<.001
Asynchronous, *N* = *[23, 32]*	4.41 (3.99, 4.83)	5.54 (5.21, 5.87)	1.14 (0.69, 1.58)	0.83	<.001
Difference	0.07	−0.26	−0.33		
p^3^	0.73	0.14	**0.15**		
Skills Scale					
Synchronous, *N* = *[434, 381]*	4.34 (4.23, 4.46)	5.74 (5.64, 5.84)	1.4 (1.27, 1.52)	1.08	<.001
Asynchronous, *N* = *[23, 32]*	4.69 (4.2, 5.17)	5.67 (5.33, 6.02)	0.98 (0.49, 1.48)	0.66	<.001
Difference	0.34	−0.07	−0.41		
p^3^	0.18	0.7	**0.11**		
Attitude Scale					
Synchronous, *N* = *[434, 381]*	5.71 (5.64, 5.77)	6.16 (6.08, 6.23)	0.45 (0.38, 0.53)	0.58	<.001
Asynchronous, *N* = *[23, 32]*	5.98 (5.69, 6.27)	6.16 (5.89, 6.43)	0.18 (−0.11, 0.47)	0.19	0.22
Difference	0.28	0.01	−0.27		
p^3^	0.07	0.96	**0.07**		

Mixed-effects linear regression with participant random intercept and time random slope with main effects for time, asynchronous delivery, and their interaction

^1^Cohen’s d standardized effect size

^2^p: *p* values for within group change over time

^3^p: *p* values for within time period between-group differences and between-group differences in the change over time (bolded)

### Usefulness and broad applicability of the session

Our post-survey included four questions assessing the usefulness and broad applicability of the session on a 7-point Likert scale. The majority of participants selected some level of agreement (i.e., slightly agree, agree, strongly agree) that the session helped them (1) meet trainees and/or faculty with whom they may want to collaborate on future work/projects (313/403 = 78%) and (2) feel connected to their motivations for pursuing a career in healthcare (351/403 = 87%)—outcomes that reflect collaboration and professional identity formation, respectively, and conceptually align with the overarching goals of advocacy training. The session also helped them (3) clarify concepts they could not learn on their own (321/403 = 80%), and (4) gain new knowledge on an advocacy topic of importance to them (343/403 = 85%).

## Discussion

Our required half-day advocacy session appeared to support trainees in developing their professional identities and acquiring foundational KSA needed to fulfill their roles as health advocates. The data analysis elucidated key strengths, highlighted areas for improvement, and provided an opportunity to reflect on next steps in making effective advocacy curricula more accessible across medical schools.

### The development of overarching knowledge, skills, and attitudes

Statistically significant improvement seen across almost all KSA domains indicates that the session as a whole was effective in achieving desired learning outcomes. This finding is notable given prior literature suggesting that advocacy skills-building is more commonly emphasized in elective rather than required courses; it supports the feasibility of integrating practical advocacy training into required UME curriculum and suggests such training can be impactful even in a short session [14]. Only two items, both pertaining to attitudes, did not reach statistical significance, which we hypothesize could be attributed to ceiling effects and possibly the notion that individuals’ attitudes towards a topic may take more time and energy to evolve than in one half-day educational session.

Students showed improvement in session-level KSAs irrespective of which two workshops they chose to attend, suggesting that the 50-minute didactic played a potentially important role in imparting key KSA or at least in establishing a foundation upon which students could develop KSA during the workshops. We also noted that, despite the ever-evolving changes in the broader sociopolitical climate, students continued to identify advocacy as essential for change pre-session and showed statistically significant increases in the belief that advocacy will be an important part of their future career moving forward post-session.

### Workshop efficacy

Nine survey questions assessed the impact of attending specific workshops (i.e., workshop-level KSAs). Statistically significant differences between attendees and non-attendees were observed in four of the nine workshop-level KSAs, meaning the workshop successfully addressed workshop-level learning goals (e.g., students who attended the research advocacy workshop indicated greater familiarity with specific research methods that promote equity and social justice than non-attendees). The five workshop-level KSAs that did not reach statistical significance still showed learning gains among all groups, which suggests that the didactic and/or other workshops likely supported learning in these areas as well.

The community engagement workshop was the only workshop in which attendees and non-attendees did not have any reliably different outcomes. Multiple student comments in the 2023 and 2024 post-surveys stated that, while the workshop conceptually illustrated the process of partnering with community organizations, it would have been more helpful to hear directly from community health workers and gain concrete tools for initiating community partnerships. These sentiments align with global literature documenting the importance of directly involving community members and organizations into the learning process, particularly through community-embedded experiential learning opportunities, and highlight a key area for improving future iterations of our community engagement workshop [34–37].

### In-person synchronous vs online asynchronous learning

Our session was designed for synchronous delivery, and the evidence suggests that synchronous in-person learning was perhaps more valuable than online asynchronous learning with between-group differences showing reliable effect at the p < 0.1 level. We hypothesize that students in the synchronous environment may have benefited from increased interactivity, collaboration with peers, and real-time feedback [38]. However, given that our assessment tool did not capture the reason for the reported between-group differences and the small sample size of asynchronous learners, this finding should be interpreted cautiously and considered preliminary.

Online asynchronous learners still showed statistically significant increases in knowledge and skills, which demonstrates that the session can still be beneficial when delivered asynchronously. Many programs, especially smaller ones, face shared barriers in implementing advocacy curricula related to variable faculty expertise, lack of protected time for session development and delivery, broader curricular constraints, and resource limitations [39–42]. The ability to deliver a session like ours asynchronously, with modifications to content and delivery as necessary, opens exciting possibilities for educators interested in providing advocacy training to their medical students.

### Distinct design principles

We intentionally designed our surveys to be more than data collection tools: they served as an opportunity for students to reflect upon their ideas about health advocacy prior to joining our session, learn briefly about the areas of advocacy we covered during the session, and reflect on key takeaways immediately after the session. As part of the post-survey, students were given space to develop a personal Specific, Measurable, Achievable, Relevant, and Time-bound (SMART) goal related to their learning in the session; these goals were sent back to them one year later to stimulate reflection upon the session and its impact during their clerkship year. While research shows that surveys can offer clear opportunities for learning and reflection, we have not seen it used much in practice and hope our use of surveys as a learning tool encourages broader use of survey-based experiential learning pedagogy in medical education [43, 44].

From a logistical standpoint, successful session implementation relied heavily upon coordinated efforts amongst a multidisciplinary team of educators and administrative support staff months in advance. Facility reservations were made thoughtfully to ensure that rooms were conducive to didactic instruction and both small- and large-group learning. Technology was utilized to facilitate participation (e.g., we created shared online documents ahead of time to seamlessly allow small groups to share ideas). We included time within FOP sessions for students to complete their sign-up and pre- and post-surveys, ensuring high survey completion rates.

### Limitations of study design and session

There were several important limitations of our study design. Similar to prior reports in the literature, assessment presented challenges [21, 22, 24]. The exclusive use of Likert-scale items across all domains limited our ability to truly measure students’ acquisition of advocacy-related KSA. Since no objective assessments were administered to verify improvements, our results are indicative of *perceptions* of changes in KSA. Thus, observed improvements in the knowledge and skills domains may reflect increased confidence or familiarity rather than true mastery of subject matter.

The survey instruments themselves had limitations. Our findings suggest that the didactic session supported students in establishing a baseline understanding of core advocacy concepts, but none of our survey questions isolated the impact of the didactic alone on KSA. Workshop-level survey questions assessed some but not all of the KSA domains. We did not pilot the surveys for feedback on usability, readability, and internal consistency, which could have improved both item clarity and the reliability of domain-specific measures. And, of course, our survey tool not being validated limits our ability to draw broadly applicable and reproducible conclusions.

While our work demonstrates that a well-developed, intentional session can support students in embracing the health advocate role and developing tools needed to become effective agents of change, we recognize that limited exposure may affect students’ overall understanding of advocacy as well as their ability to retain acquired KSA long-term. The benefits of longitudinal advocacy education over one-time interventions are well-documented in the literature and highlight an important direction for future curricular development.[22, 45, 46].

### Next steps and future considerations

The advocacy session we describe will continue to be delivered annually, with targeted modifications aimed at strengthening educational experience and learner assessment. Specifically, we plan to integrate community members and organizations early in planning process, so we can collaboratively lead a community engagement workshop that provides more concrete tools for initiating and sustaining community partnerships. We also hope to develop survey items that isolate the impact of the didactic session and more comprehensively assess workshop-level KSA to ensure that each component of the session is evaluated across all domains. Validated survey tools will strengthen our ability to measure true impact of our work.

Learning more about the long-term impact of participation in this one-time advocacy session on students and their practice of medicine – which may ultimately serve as a proxy for downstream effects on patients and communities – is another critical next step. To begin exploring this, we included reflection items in the post-survey to encourage students to think longitudinally about how the session may influence their future careers and approach to patient care. Moving forward, we hope to survey students at annual intervals to see if the improvements in KSA observed immediately after session participation have a lasting impact on identity formation and whether they translate into meaningful advocacy engagement as students advance in their training.

We plan on integrating our findings with related advocacy work at our institution to identify opportunities and strategies for more longitudinal integration of advocacy education into our undergraduate medical curriculum. Learning from best practices at other institutions, we hope to lead this charge in close collaboration with student leaders, patients, and community members to move away from hierarchical dissemination of curricula toward true co-creation [3, 10, 21, 47].

Our experience offers practical considerations for educators nationally and internationally seeking to design meaningful advocacy sessions for medical students and for leaders at accrediting organizations involved in supporting advocacy curricula. First, we underscore the importance of supportive messaging and policies around the integration of advocacy training into medical school education. Accrediting organizations must consider mandating and standardizing, rather than simply recommending, this type of formative training [12]. The integration of advocacy education within pediatrics residency programs demonstrates how accreditation mandates from the Accreditation Council for Graduate Medical Education can facilitate widespread curricular adoption [12].

At individual institutions, administrative leaders can support students, course leaders, faculty educators, and community members involved in curriculum development and dissemination through protected time and appropriate financial support [12, 22]. Most course leaders and faculty educators at our institution did not receive institutional funds or protected time to develop and deliver this session. Community partners were compensated for their time and parking using applicable grant funding. Academic institutions often capitalize on the intrinsic motivation of faculty members, and even community members, to educate and serve without providing commensurate compensation that reflects the value of these contributions. Institutional leaders can support curricular longevity, faculty engagement, and community partnerships by allocating resources for these endeavors.

We invite and encourage curriculum leaders to utilize the materials we have prepared for our didactic and workshops in an effort to nurture collaboration and decrease duplication of efforts. We are fortunate to have numerous colleagues with expertise and experience in advocacy education and session development who came together to create and disseminate the session discussed here, but we recognize that not all programs have similar resources. We hope our session will serve as a model that other institutions can adapt and implement in accordance with their unique needs, experiences, skills, and partnerships to maximize session impact and students’ learning irrespective of advocacy-specific resources. Multi-institutional replication and collaboration provide a much-needed opportunity for more rigorous dissemination, evaluation, and standardization of advocacy training efforts across institutions.

## Conclusions

Thoughtfully created advocacy sessions can empower today’s medical students with practical tools needed to care for patients in an increasingly complex healthcare system strained by worsening provider shortages, rising costs, and challenging sociopolitical pressures. As we work to promote the whole health and wellness of our patients, communities, and colleagues, it is important to recognize the role of physicians as health advocates and support impactful advocacy education that prepares trainees to engage in these efforts.

In this multi-year implementation, a required half-day advocacy session was associated with notable improvements in first-year medical students’ advocacy-related knowledge, skills, and attitudes, with the largest gains observed in knowledge and skills. These findings demonstrate that, even within a half-day, educators can contribute to early building blocks of students’ professional identity formation as health advocates and introduce elements of a toolkit to enact effective change. Limitations of these findings include reliance on self-reported outcome measures, use of survey instruments that have not yet been validated, and delivery of a one-time intervention instead of a longitudinal curriculum. Overall, these results underscore the value of integrating structured advocacy training early in medical education while also highlighting the need for validated assessment and evaluation tools, longitudinal curricular integration, and longitudinal measurement of outcomes. Future efforts should focus on strengthening assessment tools, incorporating community partnerships, and evaluating whether early gains translate into sustained engagement and impact on patient and community outcomes. Support from accrediting bodies and institutional leaders can further advance these efforts.

We hope that institutions worldwide will utilize our work to educate trainees in their programs and collaborate in gathering long-term data that will hopefully demonstrate a positive impact on physician careers and societal health outcomes.

## Supplementary Information

Supplementary Material 1

Supplementary Material 2

## Data Availability

The datasets used and/or analyzed during the current study are available from the corresponding author on reasonable request.

## Abbreviations

AMA: American Medical Association
DGSOM: David Geffen School of Medicine
FOP: Foundations of Practice
HEALS: Healer, Educator, Advocate, Leader, Scholar
KSA: Knowledge, skills, and attitudes
UCLA: University of California Los Angeles
UME: Undergraduate medical education
