# Supplementary material for: Curricular advances to develop medical students’ knowledge, skills, and attitudes in health advocacy
Source: BMC Med Educ. 2026 Jul 3;26:1256. doi: 10.1186/s12909-026-09837-w (PMC13445688; doi:10.1186/s12909-026-09837-w)
Supplement: Supplementary file 1 [file 12909_2026_9837_MOESM1_ESM.docx]

**FOP Physician Advocacy Pre-Curriculum Survey**

**Introduction [Description Text]:**

Please complete the following survey prior to next week’s advocacy session. Your responses will help us (1) better understand your background in advocacy work (no background is required though!) prior to the workshop, (2) ultimately determine whether the workshop achieved its intended goals in promoting advocacy-centered skills, knowledge, attitudes, and long-term engagement, and (3) formulate next steps in advancing advocacy education.

The data you submit as part of this survey is confidential, and the researchers will be blinded from any potential identifying information you provide. Your participation in the survey is voluntary but highly encouraged as a professional responsibility, and you have the right to withdraw your participation at any time. The results of this study will be used for scholarly purposes only. By completing this survey, you are providing your consent to participate in the advocacy survey and study. [Study information: IRB #22-001259, Humanities Curriculum for First-Year Medical Students) was certified exempt per 45 CFR 46.104 category 1.]

**Q1 [Free Text]:**

Please enter your UCLA Student ID to allow pairing of pre- and post-survey results. You will use this same ID for your post-survey.

**Q2 [Open-Ended, 500-character limit]:**

What comes to mind when you hear or think about the term *advocacy*?

**Q3 [Multiple-Choice – select all that apply] [put on new Qualtrics page]:**

I have previously heard about and/or engaged in the following forms of advocacy [choose all that apply]:

- Administrative advocacy (influencing the rules that government agencies put in place to implement laws)
- Community engagement (promoting the well-being and functioning of communities by partnering directly and collaborating with communities and community members)
- Financial advocacy (influencing the allocation and implementation of money)
- Legal advocacy (working within the legal system on behalf of another person to advocate for their rights or needs)
- Legislative advocacy (influencing laws and policies by engaging with elected officials on local, state, or federal levels)
- Media advocacy (strategically using mass media, such as television, radio, social media, etc to promote a particular cause)
- Patient advocacy in a clinical setting (bringing voice and attention to the various needs of individual patients that impact their health and well-being)
- Peer-to-peer advocacy (highlighting and promoting the needs of others who share similar challenges or experiences)
- Persuasive writing (using the written word to give voice to an issue of interest and convince others in your viewpoint)
- Professional advocacy (advocating for one’s profession often as part of a professional organization)
- Research advocacy (reducing inequity and social injustice through research)
- Other

**Q4 [7 point Matrix-Scale; put on new Qualtrics page]**

*Choose the level to which you agree or disagree with the following statements.*

1. Advocacy work is essential for change.
2. I can clearly define physician advocacy.
3. My profession makes me well-suited to be an advocate.
4. Being an advocate is an important part of who I currently am
5. I feel confident in my ability to advocate for causes I care about.
6. I know at least one way to advocate in less than 5 minutes.
7. I know the names of my state congresspeople.
8. I know the names of my federal congresspeople.
9. I know how to contact my representatives.
10. I feel comfortable contacting my representatives.
11. I know at least one professional organization with which I can collaborate on advocacy efforts.
12. Persuasive writing is a powerful tool for physician advocacy.
13. I know how to write a lede paragraph for an op-ed.
14. I can distinguish between the volunteerism model and the “upstream medicine” approach to community engagement.
15. I can identify key strategies for building community partnerships.
16. I understand conceptually how research can be used as an advocacy tool
17. I am familiar with specific research methods a researcher can use to promote equity/social justice in studies.
18. In my own life, I have experience using research findings to promote social justice.
19. Collaboration is foundational to the success of advocacy efforts.
20. Being an advocate will be an important part of my future career.
21. I have a clear understanding of how I would like to incorporate advocacy into my professional activities this coming year.

*7-Point Scale:*

- Strongly Disagree
- Disagree
- Slightly Disagree
- Neither agree nor disagree
- Slightly agree
- Agree
- Strongly Agree

**Q5 [Free-Text, 500-character limit, put on new Qualtrics page]:**

Briefly describe what you hope to gain from this advocacy session.

**Q6 [Multiple choice, one answer, put on new Qualtrics page]:**

What race or ethnicity best describes you:

- American Indian or Alaskan Native
- Asian / Pacific Islander
- Black or African American
- Hispanic
- White / Caucasian
- Multiple ethnicity / Other (please specify): _________
- I choose not to answer

**Q7 [Multiple choice, one answer]:**

Which of the following most closely describes your gender:

- Woman
- Man
- Transgender woman
- Transgender man
- Non-binary
- Agender
- My gender is not listed. My gender is: ________________
- I choose not to answer
