# Supplementary material for: Curricular advances to develop medical students’ knowledge, skills, and attitudes in health advocacy
Source: BMC Med Educ. 2026 Jul 3;26:1256. doi: 10.1186/s12909-026-09837-w (PMC13445688; doi:10.1186/s12909-026-09837-w)
Supplement: Supplementary file 2 [file 12909_2026_9837_MOESM2_ESM.docx]

**FOP Physician Advocacy Post-Curriculum Survey**

**Introduction [Description Text]:**

Please take 10 minutes to complete the following survey. Your responses will help us (1) determine whether the workshop achieved its intended goals in promoting advocacy-centered skills, knowledge, attitudes, and long-term engagement and (2) formulate next steps in advancing advocacy education.

The data you submit as part of this survey is confidential, and the researchers will be blinded from any potential identifying information you provide. As previously stated, your participation in the survey is voluntary but highly encouraged as a professional responsibility, and you have the right to withdraw your participation at any time. The results of this study will be used for scholarly purposes only. By completing this survey, you are providing your consent to participate in the advocacy survey and study. [Study information: IRB #22-001259, Humanities Curriculum for First-Year Medical Students) was certified exempt per 45 CFR 46.104 category 1.]

**Q1 [Free Text]:**

Please enter your UCLA Student ID to allow pairing of pre- and post-survey results. This is the same ID you used for your pre-survey.

**Q2 (Multiple choice – select all that apply]:**

Which two workshops did you attend today?

- Legislative advocacy
- Persuasive communication via op-ed writing
- Community engagement
- Research advocacy

**Q3 [Open-Ended, new Qualtrics page]:**

Physician advocacy is ____________________________________________________________.

**Q4 [Multiple-Choice – select all that apply, new Qualtrics page]:**

I can define the following forms of advocacy [choose all that apply]:

- Administrative advocacy
- Community engagement
- Financial advocacy
- Legal advocacy
- Legislative advocacy
- Media advocacy
- Patient advocacy in a clinical setting
- Peer-to-peer advocacy
- Persuasive writing
- Professional advocacy
- Research advocacy
- Other

**Q5 [7 point Matrix-Scale, new Qualtrics page]**

Choose the level to which you agree or disagree with the following statements.

1. Advocacy work is essential for change.
2. I can clearly define physician advocacy.
3. My profession makes me well-suited to be an advocate.
4. Being an advocate is an important part of who I currently am
5. I feel confident in my ability to advocate for causes I care about.
6. I know at least one way to advocate in less than 5 minutes.
7. I know the names of my state congresspeople.
8. I know the names of my federal congresspeople.
9. I know how to contact my representatives.
10. I feel comfortable contacting my representatives.
11. I know at least one professional organization with which I can collaborate on advocacy efforts.
12. Persuasive writing is a powerful tool for physician advocacy.
13. I know how to write a lede paragraph for an op-ed.
14. I can distinguish between the volunteerism model and the “upstream medicine” approach to community engagement.
15. I can identify key strategies for building community partnerships.
16. I understand conceptually how research can be used as an advocacy tool
17. I am familiar with specific research methods a researcher can use to promote equity/social justice in studies
18. In my own life, I have experience using research findings to promote social justice
19. Collaboration is foundational to the success of advocacy efforts.
20. Being an advocate will be an important part of my future career
21. I have a clear understanding of how I would like to incorporate advocacy into my professional activities this coming year.
22. I met trainees and/or faculty members at this workshop with whom I may want to collaborate on future work/projects.
23. This workshop helped me feel connected to my motivations for pursuing a career in healthcare.
24. This workshop clarified concepts I could not learn on my own.
25. During this workshop, I gained new knowledge on an advocacy topic of importance to me.

*7-Point Scale:*

- Strongly Disagree
- Disagree
- Slightly Disagree
- Neither agree nor disagree
- Slightly agree
- Agree
- Strongly Agree

**Q6 [Free-Text, 500-character limit, new Qualtrics page]:**

Write a SMART goal for an advocacy-related effort you want to engage in within the next 6 months (e.g., I will call my State Senator at least once in the next 3 months to discuss my thoughts on an active bill. I will commit 15 minutes every week to researching how climate change is worsening the housing crisis in Los Angeles. I will complete writing the op-ed I started writing in the workshop today by the end of 2023.)

**Q7 [Free-Text, 500-character limit]:**

What is one key takeaway from today’s advocacy session?

**Q8 [Free text, 500-character limit]:**

How do you think participation in today’s advocacy session will impact your career as a physician?

**Q9 [Free text, 500-character limit]:**

How do you think participation in today’s advocacy session will impact outcomes for the patients and communities you serve moving forward?

**Q10 [Free-Text, 500-character limit]:**

Please use this space to provide any additional comments, insights, and feedback you have.
